# A model-based head-to-head comparison of single-agent lurbinectedin in the pivotal ATLANTIS Study

**DOI:** 10.3389/fonc.2023.1152371

**Published:** 2023-06-15

**Authors:** Salvador Fudio, Laura Pérez-Ramos, Eduardo Asín-Prieto, Ali Zeaiter, Rubin Lubomirov

**Affiliations:** ^1^ Clinical Pharmacology Department, PharmaMar, S.A., Madrid, Spain; ^2^ Clinical Development and Regulatory Affairs, PharmaMar, S.A., Madrid, Spain

**Keywords:** lurbinectedin, SCLC, exposure-response, modeling, simulation, Atlantis

## Abstract

**Introduction:**

Lurbinectedin is a selective inhibitor of oncogenic transcription U.S. Food and Drug Administration (FDA)-approved for patients with relapsed small cell lung cancer (SCLC) as monotherapy at 3.2 mg/m^2^ every 3 weeks (q3wk). ATLANTIS was a phase 3 study in SCLC with lurbinectedin 2.0 mg/m^2^ plus doxorubicin 40 mg/m^2^ q3wk vs physician’s choice, with overall survival (OS) as the primary endpoint and objective response rate (ORR) as the secondary endpoint. This work aimed to dissect the contribution of lurbinectedin and doxorubicin to antitumor effects in SCLC, and to predict the efficacy of single-agent lurbinectedin at 3.2 mg/m^2^ in ATLANTIS to allow for a head-to-head comparison with the control arm.

**Methods:**

The dataset included exposure and efficacy data from 387 patients with relapsed SCLC (ATLANTIS, n=288; study B-005, n=99). Patients in the ATLANTIS control arm (n=289) were used for comparison. Unbound plasma lurbinectedin area under the concentration-time curve (AUC*_u_*) and total plasma doxorubicin area under the concentration-time curve (AUC*_DOX_*) were used as exposure metrics. Univariate and multivariate analyses were conducted to determine the best predictors and predictive model for OS and ORR. OS baseline hazard was best described by a log-logistic distribution, with chemotherapy-free interval (CTFI), lactate dehydrogenase, albumin, brain metastases, neutrophils/lymphocytes ratio, AUC*_u_*, and the interaction between AUC*_u_* and AUC*_DOX_* as predictors. Effect of AUC*_u_* on ORR best fitted to a sigmoid-maximal response (*E_max_*) logistic model, where *E_max_* was dependent on CTFI.

**Results:**

Head-to-head comparisons with predicted 3.2 mg/m^2^ lurbinectedin resulted in a positive outcome in ATLANTIS, with hazard ratio (95% prediction intervals [95% PI]) for OS of 0.54 (0.41, 0.72), and odds ratio (95% PI) for ORR of 0.35 (0.25, 0.5).

**Conclusion:**

These results support the superiority of lurbinectedin monotherapy for relapsed SCLC over other approved therapies.

## Introduction

Small cell lung cancer (SCLC) is an aggressive disease, based on a 7% five-year survival, that accounts for approximately 15% of all lung cancers, thus killing an estimated 250,000 people worldwide yearly (1, 2). SCLC is a transcription-addicted disease, with high levels of dysregulated transcription factors that contribute to tumor initiation and progression (3).

Lurbinectedin (Zepzelca^®^) is a synthetic marine-derived anticancer agent that acts as a selective inhibitor of oncogenic transcription. It binds preferentially to guanines located in the GC-rich regulatory areas of DNA gene promoters (4, 5). By preventing binding of transcription factors to their recognition sequences, lurbinectedin inhibits oncogenic transcription and leads to tumor cell apoptosis (6). Lurbinectedin also affects the tumor microenvironment landscape by inhibiting activated transcription in tumor-associated macrophages (7).

An intensive early phase of lurbinectedin clinical development, involving pharmacometric analyses (8), led to the selection of 3.2 mg/m² every 3 weeks (q3wk) as the dose regimen for lurbinectedin as monotherapy.

Lurbinectedin was also investigated in combination with doxorubicin in phase 1b study A-003 (NCT01970540), on the basis of the preclinical evidence of potential synergistic effects (9). At the final recommended dose regimen of 2.0 mg/m² lurbinectedin q3wk and 40 mg/m² doxorubicin q3wk for second-line SCLC, objective response rate (ORR) by investigator assessment (IA) was 36% (95% confidence interval [CI]: 18.6, 55.9), with a median (95% CI) overall survival (OS) of 7.9 (4.2, 11.5) months. These findings formed the rationale for ATLANTIS (NCT02566993), a phase 3 randomized study in second-line SCLC with lurbinectedin 2.0 mg/m² plus doxorubicin 40 mg/m² q3wk as the experimental arm. For the control arm, doxorubicin as monotherapy could not be selected as this is not a standard of care in this setting. Therefore, the control arm consisted of physician’s choice between CAV (cyclophosphamide 1000 mg/m² plus doxorubicin 45 mg/m² plus vincristine 2.0 mg) and topotecan 0.75–1.50 mg/m², days 1–5, q3wk.

In the meantime, a phase 2 basket trial with single agent lurbinectedin (study B-005; NCT02454972) included, among nine difficult to treat cancer types, a cohort of 105 patients with second-line SCLC. In this cohort, ORR by IA was 35.2% (95% CI: 26.2, 45.2), median (95% CI) duration of response (DoR) was 5.3 (4.1, 6.4) months, and median (95% CI) OS was 9.3 (6.3, 11.8) months (10). Based on these results, in June 2020 the U.S. Food and Drug Administration (FDA) granted accelerated approval to lurbinectedin as a single agent at 3.2 mg/m² q3wk for patients with metastatic SCLC with disease progression on or after platinum-based chemotherapy, with ATLANTIS as phase 3 randomized confirmatory study. The Exposure-Response (E-R) relationship between single-agent lurbinectedin exposure and efficacy and safety endpoints observed in this study ratified the adequacy of the 3.2 mg/m² q3wk dose regimen (11).

ATLANTIS did not meet its primary objective of superiority in OS, with a hazard ratio (HR) of 0.97 (95% CI: 0.82, 1.15) and median OS (95% CI) of 8.6 (7.1, 9.4) months in the experimental arm vs 7.6 (6.6, 8.2) months in the control arm (12), and thus it could not serve as a confirmation of the activity of lurbinectedin observed in study B-005. However, since ATLANTIS included neither lurbinectedin alone nor doxorubicin alone, a direct comparison of the efficacy of lurbinectedin vs the standard of care in ATLANTIS was not feasible.

A new phase 3 confirmatory study (LAGOON; NCT05153239) in second-line SCLC, including 3.2 mg/m² single agent lurbinectedin in the experimental arm is ongoing, and will provide direct evidence on whether lurbinectedin is superior to the standard of care.

The pharmacometric-based E-R analysis presented herein leverages ATLANTIS to anticipate the performance of single agent lurbinectedin in a phase 3 setting. E-R analysis can provide supportive evidence to address complex questions regarding efficacy, such as the estimation of the contribution of an individual drug to a combination (13). In this sense, the aim of this E-R analysis was two-fold: 1) to estimate the contribution of lurbinectedin in OS and ORR when added to doxorubicin, and 2) to predict the efficacy of single-agent lurbinectedin at 3.2 mg/m^2^ in the same population of ATLANTIS to allow a head-to-head comparison with the control arm.

## Methods

### Patients and data

This E-R analysis was conducted based on the efficacy and pharmacokinetic (PK) data from patients treated with lurbinectedin as a single agent in the SCLC cohort of study B-005, and with lurbinectedin in combination with doxorubicin in ATLANTIS. Efficacy data from patients in the control arm of ATLANTIS were used to perform the head-to-head comparisons.

Study B-005 was a single-arm, multicenter, open-label, phase 2 basket study in patients with relapsed SCLC and eight other difficult-to-treat tumors. Patients with brain metastases were not allowed. Patients received 3.2 mg/m² lurbinectedin as a single agent during a 1-h intravenous (IV) infusion q3wk. The primary efficacy endpoint was confirmed ORR; secondary endpoints included OS.

ATLANTIS was a randomized, multicenter, open-label, phase 3 study in patients with relapsed SCLC and with a minimum chemotherapy-free interval (CTFI) of 30 days. Patients were randomized 1:1 to receive lurbinectedin 2.0 mg/m² plus doxorubicin 40 mg/m² q3wk, or either CAV or topotecan q3wk in a control arm. The primary endpoint was OS; ORR was considered as a secondary endpoint.

Details of PK sampling times, plasma concentration measurement, and OS and ORR definition are provided in the **Supplementary Material**.

The protocols and informed consent forms of study B-005 and ATLANTIS were approved by the institutional review boards, and written informed consents were provided by all patients before any study-related procedures were performed.

### Computer software

Nonlinear mixed-effect modeling (NONMEM) of concentration-time data, exposure metrics derivation, and E-R analysis of ORR were performed using NONMEM v.7.3.0 (ICON, Ellicott City, MD). Stochastic Approximation Expectation-Maximization (SAEM) and Monte Carlo Importance Sampling Expectation-Maximization (IMP) were used as estimation methods. Models for OS were performed using R, v.4.0.1 (Comprehensive R Archive Network, http://cran.r-project.org).

### PK analyses and exposure metrics

As for previous E-R analyses of efficacy and safety with lurbinectedin (11), unbound plasma exposure (AUC*_u_*) during cycle 1 was selected as the exposure metric. A reference population PK (PopPK) model (14) was updated with PK data of ATLANTIS, to provide empirical Bayesian estimates on individual clearance (CL). These were transformed to AUC*_u_* using individual alpha-1-acid glycoprotein (AAG), albumin, and total dose as described elsewhere (11).

Total plasma exposure (AUC*_DOX_*) during cycle 1 was selected as the exposure metric of doxorubicin. A published PopPK model (15) was used as reference model and updated accordingly to fit the doxorubicin-time profiles observed in ATLANTIS and to obtain individual estimates of AUC*_DOX_*.

### E-R analyses

Exploratory analyses were conducted to detect any potential relationship between the exposure metrics, and OS and ORR, as well as the prognostic factors identified, in the overall population and stratified by study/arm.

Univariate Kaplan-Meier regression and logistic regression analyses were conducted to explore the influence of relevant prognostic factors and exposure metrics on OS and ORR. Multivariate Cox regression and parametric analyses (i.e., Weibull, Gompertz exponential, lognormal, log-logistic, Gaussian) and multivariate logistic regression analyses were conducted to simultaneously incorporate the effect of AUC*_u_* and AUC*_DOX_* as continuous variables, and the significant factors identified, and to evaluate the relative contribution of each of them into the model. The impact of AUC*_u_* and AUC*_DOX_* on OS, after adjusting for prognostic factors, was assessed by the HR or the Acceleration Factor (AF) for parametric models such as log-logistic, and their 95% CI. *P* values as well as change in Akaike information criterion (AIC) and -2loglikelihood (-2LL) between rival comparative models were used for model comparison and selection. For OS, calibration plots and predictive error at different time points plots were also used for the diagnosis of each candidate model. For ORR, accuracy, precision, recall, and F1-score were used for the evaluation of each model performance.

Dataset splitting, model training, and model testing were performed to validate the final models and to test their predictive power. For this end, visual predictive checks (VPCs) were depicted, where 95% prediction intervals (PIs) derived from 50 replicates of the predicted values at each time point (month) were compared with observed data in a training and a test dataset.

### Simulation of OS and ORR

Upon validation, the predictive ability of the final models was tested. Bootstrap resampling (250 subsamples) was generated to assess the uncertainty associated with predicted and observed OS and ORR. Therefore, a total of 250 comparisons with each fitted bootstrap replicate (median of predicted and observed OS and ORR, and the corresponding relative difference with the 95% CI) were computed and then summarized across replicates.

Finally, predicted efficacy with two alternative regimens was compared with observed efficacy in ATLANTIS: 1) single-agent 2.0 mg/m² lurbinectedin vs experimental (lurbinectedin 2.0 mg/m² plus doxorubicin) and the CAV arm, aimed at isolating the effect of lurbinectedin and doxorubicin in the experimental arm; 2) single-agent 3.2 mg/m² lurbinectedin vs the control arm (model-based head-to-head comparison).

## Results

### Participants

Unbound lurbinectedin exposure data (AUC*_u_*) and efficacy data were available from 387 patients with SCLC from study B-005 (n=99) and the experimental arm of ATLANTIS (n=288). Efficacy data from the control arm of ATLANTIS (n=289) with patients treated with CAV (n=168) or topotecan (n=121) were used to perform the head-to-head comparisons.

### Population PK models

The updated model of lurbinectedin used 10,847 total plasma concentrations from 1172 patients treated in 11 phase 1–3 clinical studies. An open, 3-compartment linear disposition model parameterized by total plasma elimination CL, apparent volumes of distribution of the central, shallow, and deep peripheral compartments (V1, V2, and V3, respectively) and two intercompartmental distribution clearances (Q2 and Q3) with estimated inter-individual variability (IIV) in CL, V1, V3, Q3, V2, and residual variability (RV), and with IIV correlation between Q3 and V3. The parameters estimates and bootstrap, including the statistically significant covariate effects on the model parameters, are presented in **Table S1**.

The doxorubicin model taken from the literature to describe the PK of doxorubicin and its metabolite doxorubicinol was an open, 4-compartment linear disposition model parameterized in total plasma elimination clearance of doxorubicin (CL*_DOX_*), apparent volumes of distribution of the central, deep, and shallow peripheral compartments of doxorubicin (V4, V7, and V8, respectively), two intercompartmental distribution clearances of doxorubicin (Q7 and Q8), total plasma elimination clearance of doxorubicinol (CL*_M_*), and apparent volume of distribution of the central compartment of doxorubicinol (V5). IIV was estimated for CL*_DOX_*, V5, V7, Q7, CL*_M_*, and RV of doxorubicin and doxorubicinol (**Table S2**).

### Exploratory analyses

A description of baseline characteristics, such as age, CTFI, presence of brain metastases, body surface area (BSA), albumin, AAG, C-reactant protein (CRP), lactate dehydrogenase (LDH), neutrophils/lymphocytes (NL) ratio and platelets/lymphocytes (PL) ratio, are shown in **Table 1** by study/arm. No differences were observed among studies, except for CTFI (mean [standard deviation]: 116.6 [90.9] days vs 157.9 [134.0] days) and presence of brain metastases (number [%]: 0 [0.0] vs 45 [15.6]) in study B-005 and the ATLANTIS experimental arm, respectively, due to differences in exclusion criteria (i.e., no brain metastasis in study B-005 and no patients with refractory disease [CTFI<30 days] in ATLANTIS).

**Table 1:** Summary of demographic and baseline characteristics of patients by study/arm.

	Study B-005	ATLANTIS				TOTAL
Covariate, mean (SD)	LRB 3.2 mg/m²	LRB 2.0 mg/m² + DOX	CAV or Topotecan	CAV	Topotecan	
*n*	99	288	289	168	121	676
Age, years	61.0 (9.6)	63.0 (8.3)	62.8 (7.6)	62.9 (8.1)	62.7 (7.0)	62.6 (8.2)
CTFI, days	116.6 (90.9)	157.9 (134.0)	153.5 (118.8)	153.1 (118.0)	154.1 (120.4)	150.0 (122.7)
BSA, m²	1.8 (0.2)	1.8 (0.2)	1.9 (0.2)	1.8 (0.2)	1.9 (0.2)	1.8 (0.2)
Albumin, g/dL	4.0 (0.5)	4.1 (0.4)	4.1 (0.4)	4.2 (0.4)	4.1 (0.5)	4.1 (0.4)
AAG, mg/dL^a^	129.3 (45.1)	129.6 (50.3)	–	–	–	129.5 (49.0)
CRP, mg/L^b^	–	23.6 (43.0)	34.0 (146.4)	32.1 (125.6)	36.5 (172.0)	28.8 (108.3)
LDH, UI/L	402.9 (355.3)	371.6 (402.1)	340.4 (223.6)	353.2 (233.0)	322.8 (209.6)	362.9 (330.1)
NL ratio	5.4 (4.6)	5.3 (4.8)	4.9 (3.9)	4.6 (3.2)	5.3 (4.6)	5.1 (4.4)
PL ratio	242.0 (129.9)	252.3 (212.8)	234.6 (149.9)	227.7 (139.2)	244.2 (163.7)	243.2 (177.0)
Brain metastases, *n* (%)	0 (0.0)	45 (15.6)	46 (15.9)	24 (14.3)	22 (18.2)	91 (13.5)

AAG, alpha-1-acid glycoprotein; BSA, body surface area; CAV, cyclophosphamide plus doxorubicin plus vincristine; CRP, C-reactant protein; CTFI, chemotherapy-free interval; DOX, doxorubicin; LDH, lactate dehydrogenase; LRB, lurbinectedin; NL, neutrophils/lymphocytes; PL, platelets/lymphocytes; SD, standard deviation.

a AAG was not collected in the control arm of study ATLANTIS.

b CRP was not collected in study B-005.

No large differences in lurbinectedin CL between studies were observed. Baseline characteristics were balanced among quartiles of AUC*_u_* and AUC*_DOX_* and when stratified by CTFI. Baseline characteristics were also evenly distributed between the experimental and the control arm of ATLANTIS in patients with resistant (CTFI<90 days) and sensitive (CTFI ≥90) disease, in light of the absence of statistically significant *P* values (< 0.05), which reassures the validity of the comparisons described later.

Therefore, the exploratory analyses indicated that the prognostic factor distribution across study/arms, quartiles of AUC*_u_* and AUC*_DOX_*, and CTFI groups were well balanced, and the minor potential imbalances that may exist should be controlled by the multivariate analyses.

Finally, a summary of efficacy metrics for all patients by study/arm and CTFI is described in **Table 2**. As expected, patients with sensitive and very sensitive disease had better outcome than those with resistant disease in all study/arms.

**Table 2:** Summary of efficacy endpoints by study/arm and CTFI.

	Study B-005	ATLANTIS			
	LRB 3.2 mg/m²	LRB 2.0 mg/m² +DOX	CAV or Topotecan	CAV	Topotecan
*All patients (N)*	99	288	289	168	121
OS					
Events	91	252	248	148	100
Median (95% CI)	8.8 (6.6, 11.8)	8.6 (7.6, 9.6)	7.6 (6.6, 8.3)	7.6 (6.1, 8.2)	7.9 (6.6, 10.3)
ORR					
% (95% CI)	30.3 (21.3, 39.4)	33.0 (27.6, 38.4)	31.5 (26.1, 36.8)	31.0 (24.0, 37.9)	32.3 (23.9, 40.6)
*Resistant* *(CTFI<90) (N)*	42	90	92	53	39
OS					
Events	40	88	85	49	36
Median (95% CI)	5.0 (4.1, 7.6)	5.7 (4.3, 6.8)	5.3 (4.4, 6.4)	5.9 (5.0, 7.8)	5.0 (3.5, 6.6)
ORR					
% (95% CI)	11.9 (2.1, 21.7)	21.1 (12.7, 29.5)	20.7 (12.4, 28.9)	24.5 (12.9, 36.1)	15.4 (4.1, 26.7)
*Sensitive* *(CTFI ≥180) (N)*	57	198	197	115	82
OS					
Events	51	164	163	99	64
Median (95% CI)	11.8 (9.7, 15.8)	10.3 (9.1, 11.8)	8.7 (7.8, 10.2)	7.9 (7.3, 9.5)	11.2 (8.2, 13.9)
ORR					
% (95% CI)	43.9 (31.0, 56.7)	38.4 (31.6, 45.2)	36.5 (29.8, 43.3)	33.9 (25.3, 42.6)	40.2 (29.6, 50.9)

CAV, cyclophosphamide plus doxorubicin plus vincristine; CI, confidence interval; CTFI, chemotherapy-free interval; DOX, doxorubicin; LRB, lurbinectedin; ORR, objective response rate; OS, overall survival.

### E-R analysis of OS

**Figure 1:**
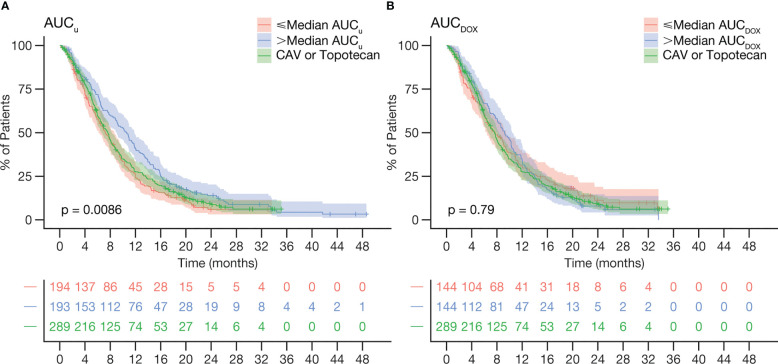
Kaplan-Meier curves of OS stratified by **(A)** lurbinectedin AUC*_u_* and **(B)** doxorubicin AUC*_DOX_*, relative to the control arm. Curves of lurbinectedin AUC*_u_* pooled data from study B-005(≤median AUC*_u_* n=27, >median AUC*_u_* n=72) and ATLANTIS (≤median AUC*_u_* n=167, >median AUC*_u_* n=121). *P*-values indicate whether there are differences in median OS from the three curves. AUC*_DOX_*, total plasma doxorubicin area under the concentration-time curve; AUC*_u_*, unbound plasma lurbinectedin area under the concentration-time curve; CAV, cyclophosphamide plus doxorubicin plus vincristine; OS, overall survival.

Univariate Cox regression analyses found statistically significant associations between OS and CTFI (<90 vs ≥90 days), AAG, albumin, LDH, CNS metastases (absence vs presence), NL ratio and PL ratio (**Supplementary Material**, **Figure S1**). AUC*_u_* showed a clear positive trend of increasing OS, especially at higher values than the median (1063.7 ng·h/L) (**Figure 1A**). On the other hand, no clear trend of improvement by AUC*_DOX_* became apparent (**Figure 1B**).

Based on these predictors for survival status, multivariate models were developed. According to AIC and -2LL, a model including CTFI, LDH as continuous log-transformed [*log*(LDH)], NL ratio, brain metastases, AUC*_u_*, AUC*_DOX_*, and interaction between AUC*_u_* and AUC*_DOX_* (AUC*_u_* ·AUC*_DOX_*) showed the best fit. Of note, although AUC*_DOX_* did not show a significant effect, AUC*_u_* ·AUC*_DOX_* did, thus involving the need to keep AUC*_DOX_* in the final model to retain AUC*_u_* ·AUC*_DOX_*.

Based on visual inspection of calibration plots and prediction error plots (**Figures S2**, **S3**), OS baseline hazard was best described by a log-logistic distribution with a shape parameter *p*, and a scale parameter *λ*. Besides, the proportional hazards assumption was not satisfied, thus the log-logistic was selected as the final model.

**Figure 2:**
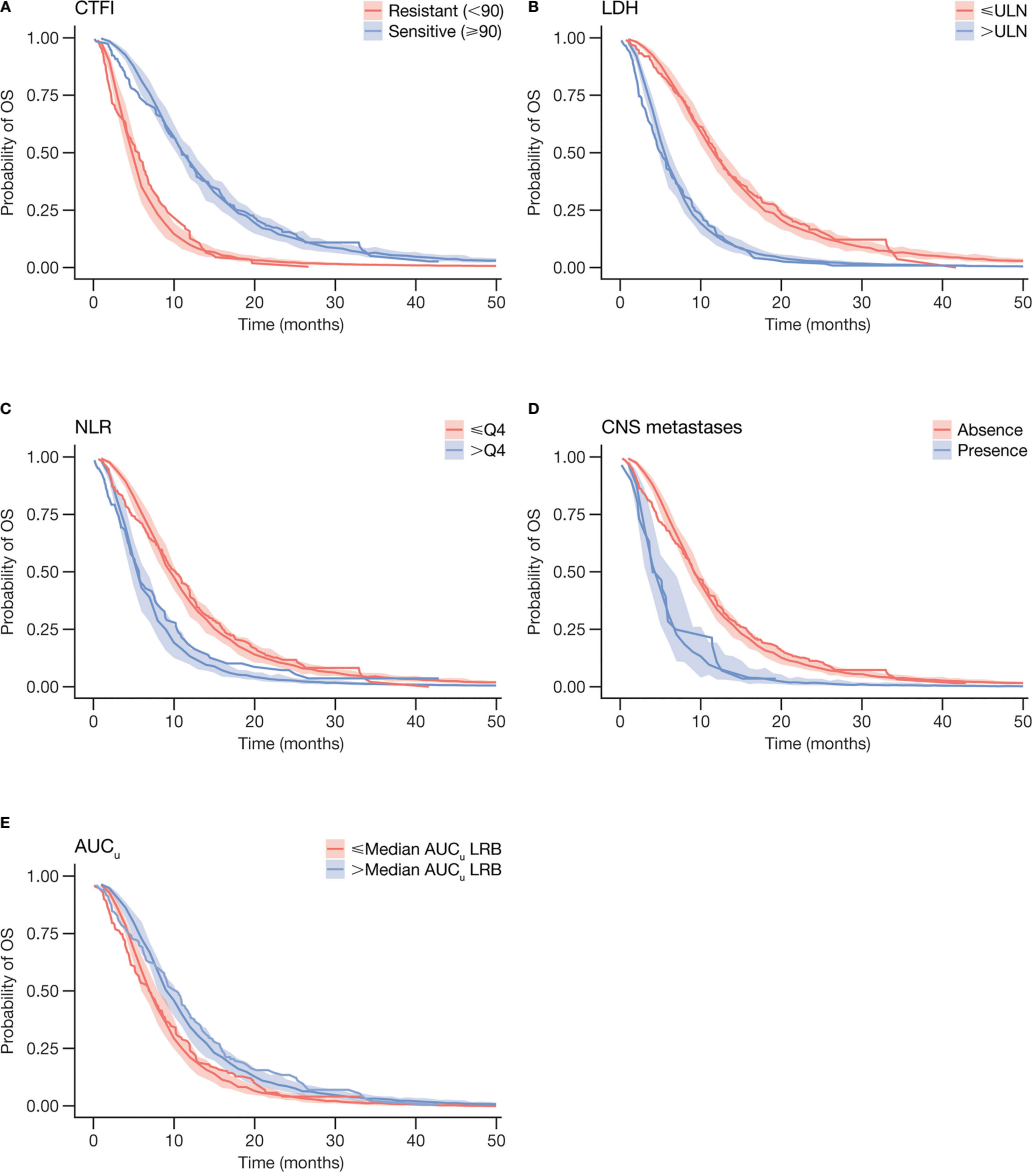
Visual predictive checks of final log-logistic OS model and Kaplan-Meier curves for OS by prognostic factors **(A)** CTFI, **(B)** LDH, **(C)** NLR, and **(D)** CNS metastases, and by **(E)** AUC*_u_*. Step-wise lines represent observed data. Shaded areas represent 95% prediction intervals derived from model simulation. AUC*_u_*, unbound plasma lurbinectedin area under the concentration-time curve; CNS, central nervous system; CTFI, chemotherapy-free interval; LDH, lactate dehydrogenase; LRB, lurbinectedin; NLR, neutrophil to lymphocyte ratio; OS, overall survival; NLR, neutrophils/lymphocytes ratio; Q4, highest NLR quartile; ULN, upper limit of normality.

VPCs, stratified by prognostic factors and AUC*_u_*, showed the goodness of fit in the training set, as well as the predictive power of the test set (**Figure 2**), thus being adequate to validate the final OS model.

The hazard function of the final OS model is described by Equation 1.


(1) $$h\left(t\right)=\frac{\lambda pt^{p-1}}{1+\lambda t^{p}}\cdot e^{\alpha_{0}+\alpha_{n}X}$$

assuming time *t*~log-logistic (*λ, p*), α are the model coefficients describing the effect of corresponding risk factor on hazard, *X* is the covariate matrix, and *n* is the number of model covariates. Acceleration factors for *λ*, *p*, and each covariate, and their corresponding 95% CIs are presented in **Table 3**. Shape parameter *p* was estimated to 2.4 (*p* > 1), meaning that the hazard, in the absence of changes in retained predictors, increased to a maximum point, and then decreased over time.

**Table 3:** Parameters of the final log-logistic OS model and the final ORR model.

Parameter (OS model)	Acceleration factor (95% CI)
p	2.4
λ	294.2
Covariates	
CTFI group (≥90)	1.9 (1.6, 2.3)
log(LDH)	0.5 (0.4, 0.6)
NL ratio	0.9 (0.9, 0.9)
Brain metastases	0.6 (0.4, 0.8)
AUC_u_ (ng·h/L)	1.4 (1.0, 1.8)
AUC_DOX_ (µg·h/L)	1.2 (0.9, 1.5)
AUC_u_·AUC_DOX_	0.8 (0.7, 0.9)
**Parameter (ORR model)**	Estimate (RSE%)
E_max_ resistant	8.5 (5.9)
E_max_ sensitive	10.8 (3.9)
EC_50_ (ng·h/L)	877 (7.7)

p, shape parameter of the log-logistic baseline hazard model; λ, scale parameter of the log-logistic baseline hazard model; AUC_DOX_, total plasma doxorubicin area under the concentration-time curve; AUC_u_, unbound plasma lurbinectedin area under the concentration-time curve; AUC_u_ ·AUC_DOX_, interaction between AUC_u_ and AUC_DOX_; CI, confidence interval; CTFI, chemotherapy-free interval; EC_50_, half maximal effective concentration; E_max_, maximal response; LDH, lactate dehydrogenase; NL, neutrophils/lymphocytes; ORR, objective response rate; OS, overall survival; RSE, relative standard error.

### Simulations of OS

Predicted median (and bootstrap 95% PI) OS was 8.2 (7.4, 9.3) months in the experimental arm of ATLANTIS, similar to observed median (95% CI) OS of 8.6 (7.6, 9.6) months in the trial, thus confirming the predictive ability of the final model.

Predicted OS (95% PI) with 2.0 mg/m^2^ lurbinectedin as a single agent in the experimental arm was 8.3 (7.5, 9.7) months, being comparable (HR: 0.96 [0.73, 1.31]) to the observed OS in the trial, although higher (HR: 0.78 [0.54, 0.97]) than in the CAV arm (7.6 [6.1, 8.2]), which suggests a marginal contribution of doxorubicin to the overall effect of the experimental arm.

**Figure 3:**
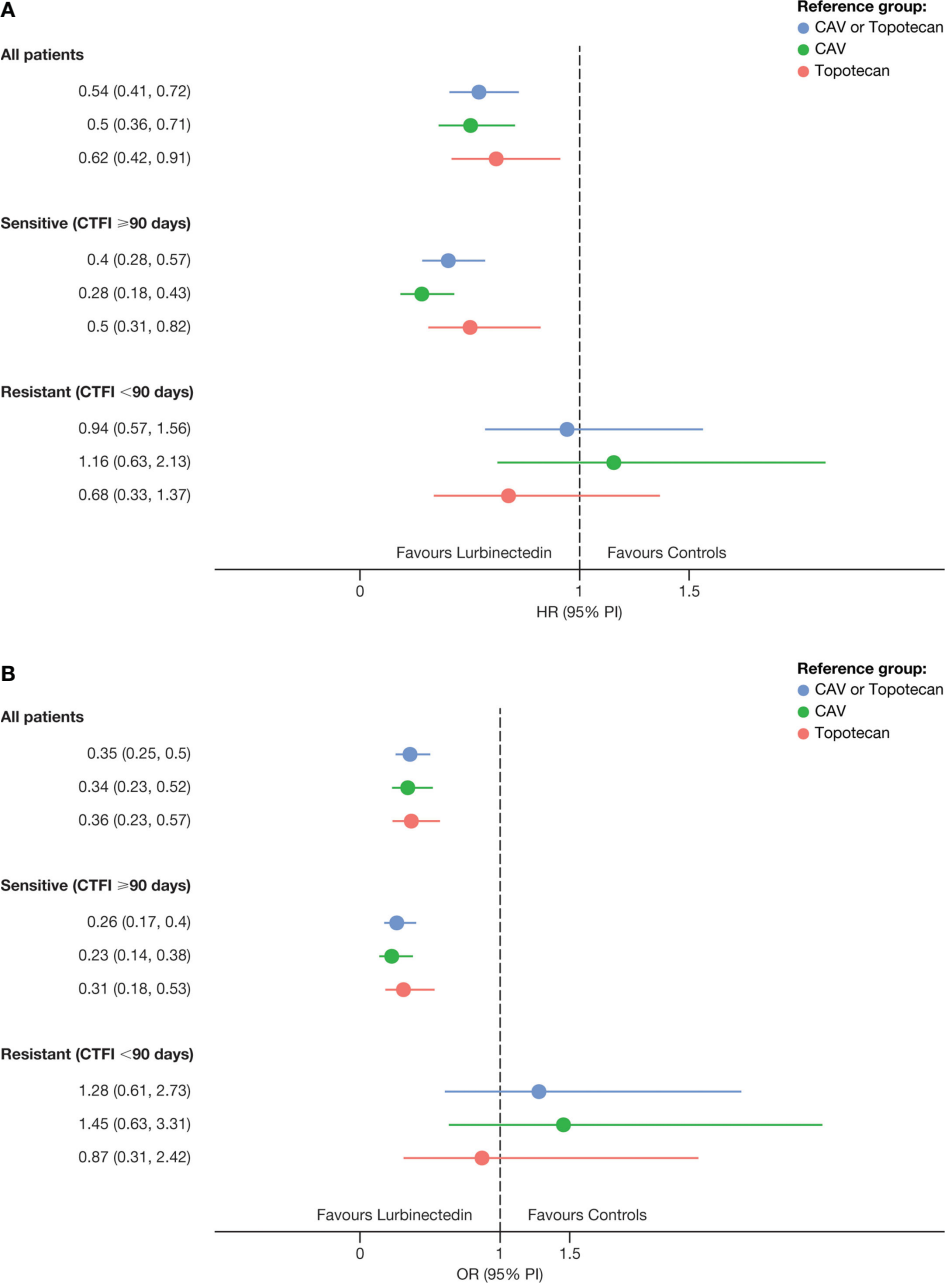
Hazard ratios for **(A)** OS and odds ratios for **(B)** ORR of predicted efficacy with lurbinectedin 3.2 mg/m² as a single agent in ATLANTIS vs control arm. CAV, cyclophosphamide plus doxorubicin plus vincristine; CTFI, chemotherapy-free interval; HR, Hazard ratio; OR, odds ratio; ORR, overall response rate; OS, overall survival; PI, prediction interval.

Predicted OS (95% PI) with 3.2 mg/m^2^ lurbinectedin as a single agent was 9.7 (9.1, 11.5) months. When compared with observed OS in the control arm (7.6 [6.6, 8.3]) months (**Table 2**), the relative difference (HR: 0.54 [0.41, 0.72]) reached statistical significance, favoring lurbinectedin. **Figure 3A** depicts the results of this head-to-head comparison.

In patients with sensitive disease (CTFI ≥90 days), predicted OS (95% PI) was 14.1 (12.3, 15.2) months, while observed OS in the control arm was 8.7 (7.7, 10.1) months (HR: 0.4 [0.28, 0.57]) (**Figure 3A**). In patients with very sensitive disease (CTFI ≥180 days), predicted OS was 16.7 (15.7, 18.5) months, leading to an even larger difference (HR: 0.28 [0.16, 0.49]) with the control arm (9.8 [7.6, 13.7] months) (**Figure S4**). In contrast, in patients with resistant disease, a predicted OS of 5.6 (0.9, 1.5) months did not show superiority over the observed OS of 5.3 (4.4, 6.4) months in that patient subgroup of the control arm (HR: 0.94 [0.57, 1.56]).

### E-R analysis of ORR

Univariate logistic regression analyses with ORR found similar associated predictors to those detected for OS. In a multivariate model, CTFI, *log*(LDH) and AUC*_u_* showed a statistically significant relationship with ORR, while AUC*_DOX_* and AUC*_u_* ·AUC*_DOX_* did not, thus indicating that the contribution of doxorubicin and its interaction with lurbinectedin to the overall activity was marginal. On the other hand, the predictive performance of this multivariate model showed similar accuracy (59.4%), and F1-score (51.1%) than a previous model of ORR for single-agent lurbinectedin (11). Therefore, this model, consisting of a sigmoid-*E_max_* model for AUC*_u_*, where CTFI modified the *E_max_* parameter (Equation 2), was selected as the final predictive model for ORR.


(2) $$logit\;\left(ORR\right)=\;-10+\frac{E_{max}\;\times AUC_{u}^{10}\;}{\;EC_{50}^{10}\;+\;AUC_{u}^{10}}$$

where EC_50_ was the AUC*_u_* that provided half *E_max_* on ORR. Parameter estimates with relative standard errors (RSEs) in the final model are shown in **Table 3**.

### Simulations of ORR

**Figure 4:**
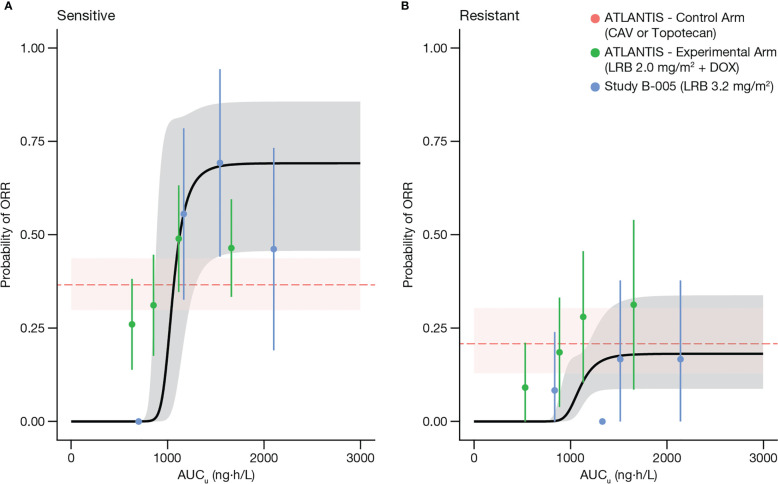
Visual predictive check of the model for ORR, stratified by patients with sensitive disease CTFI≥90 days **(A)**, and with resistant disease or CTFI<90 days **(B)**. Solid dots represent the proportion of responders grouped by quartiles of AUC*_u_* and plotted at the median AUC*_u_* for each quartile, in sensitive (left panel) and resistant (right panel) patients. Bars represent the 95% CI for the proportion of each quartile. Black curve and shaded gray area represent predicted values and 95% PI of model predicted ORR, respectively. Horizontal dashed red line and shaded pink area correspond to median and 95% CI of observed ORR of the control arm (CAV or topotecan) of ATLANTIS. AUC*_u_*, unbound plasma lurbinectedin area under the concentration-time curve; CAV, cyclophosphamide plus doxorubicin plus vincristine; CTFI, chemotherapy-free interval; DOX, doxorubicin; LRB, lurbinectedin; ORR, objective response rate.

**Figure 4** depicts VPCs by CTFI (<90 days and ≥90 days), overlapping model-predicted ORR probability, and observed ORR with single-agent lurbinectedin 3.2 mg/m^2^ (study B-005) and with lurbinectedin 2.0 mg/m^2^ plus doxorubicin (ATLANTIS), grouped by quartiles of AUC*_u_*. Of note, patients with sensitive disease (CTFI ≥90 days) in ATLANTIS showed a similar ORR (95% PI) at the lowest AUC*_u_* quartile (26% [16, 35]) to that of the CAV arm (33.9% [25.3, 42.6]).

Predicted ORR (95% PI) with 2.0 mg/m^2^ lurbinectedin as a single agent was 27.9% (22.7, 33.5) and was somewhat lower than the observed ORR of 31.7% (26.3, 37.5), although no statistically significant differences were observed (odds ratio [OR]: 1.22 [0.84, 1.78]), indicating the predictive ability of the model.

Finally, predicted ORR (95% PI) for patients in the experimental arm of ATLANTIS receiving 3.2 mg/m^2^ lurbinectedin as a single agent was 56.4% (50.4, 62.3), being superior (OR: 0.35 [0.25, 0.50)] than the observed ORR in the control arm, 31.7% (26.3, 37.5) (**Figure 3B**).

## Discussion

Pivotal studies for combination therapies usually consist of a treatment added on to the standard of care vs the standard of care alone. However, in a recent review of FDA-approved combinations for hemato-oncology, 10% of pivotal randomized studies, mainly involving chemotherapy agents but also targeted agents, didn’t follow this pattern (16), making the assessment of the contribution of each agent to the overall effect challenging.

In ATLANTIS, lurbinectedin was given at a 2.0 mg/m² dose in combination with doxorubicin at 40 mg/m^2^. In the control arm, doxorubicin was given at 45 mg/m^2^ as part of the triplet CAV. Therefore, the contribution of doxorubicin could not be isolated. On the other hand, the dose of lurbinectedin in the experimental arm was lower than the approved single-agent regimen (3.2 mg/m^2^), compromising indirect comparisons with historical data.

The present E-R analyses aimed to derive the effect size of lurbinectedin, to estimate its efficacy as a single agent at the approved 3.2 mg/m² dose, in a phase 3 randomized and controlled setting. To enable this, predictive models were built by pooling the phase 2 study B-005 (lurbinectedin at 3.2 mg/m² as a single agent) and ATLANTIS, so that they were suitable to predict the efficacy of lurbinectedin either as a single agent or in combination with doxorubicin at a broader range of doses (from 2.0–3.2 mg/m²).

PopPK models of lurbinectedin (14) and doxorubicin/doxorubicinol (15) were updated with the ATLANTIS data. A clinically meaningful effect of co-administration of doxorubicin on the CL (4.1% reduction, 90% CI: [-10.5, 2.4]) and CL*_u_* (7.5% reduction, [-13.3, -1.7]) of lurbinectedin was ruled out. This finding was consistent with the covariate assessment conducted in the initial PopPK model with data from single-agent studies and combination program, including doxorubicin. On the other hand, population estimates of doxorubicin and doxorubicinol CLs were 33.2% and 43.4% lower than historical reference values. Therefore, the effect of lurbinectedin exposure on the PK of these analytes cannot be ruled out.

OS and ORR model development included individual estimates of lurbinectedin and doxorubicin exposure (i.e., AUC*_u_* and AUC*_DOX_*, respectively), while doxorubicinol exposure, although available, was not included as it is not relevant from an efficacy standpoint.

Overall, ATLANTIS exposure and efficacy data confirmed the findings from the previous E-R analysis of study B-005 (11). Despite study B-005 only explored a single dose level of lurbinectedin, the relationship between exposure and ORR could be characterized. Maximal ORR in patients with sensitive and resistant disease occurred with AUC*_u_* lower than the median AUC*_u_* achieved with 3.2 mg/m², thus supporting the adequacy of this dose level. Regarding OS, patients at the lowest quartile of AUC*_u_* showed significantly shorter OS than patients at the highest quartiles, for both sensitive and resistant disease. Besides, multivariate Cox regression analyses including CTFI and AUC*_u_* as covariates showed that the risk of death was 4.6-fold higher for resistant patients, and every increase of AUC*_u_* (µg·h/L) unit decreased the risk of death by 2.5-fold (11).

In this E-R analysis, a new model for OS pooling E-R data from study B-005 and ATLANTIS was developed and validated, where relevant prognostic factors as well as exposure were shown to be related to variability in OS among patients with SCLC treated with lurbinectedin. CTFI, LDH, and brain metastases, well-known covariates associated with survival outcome in SCLC patients, accounted for a considerable proportion of explained variability. NL ratio, widely used in clinical practice as a surrogate marker of chronic inflammation (17), was also a predictor of shorter survival, as anticipated.

Lurbinectedin AUC*_u_* was retained in the model as a significant predictor, thus confirming its activity in patients with relapsed SCLC and allowing the derivation of the effect size of lurbinectedin on OS. In contrast, despite a trend observed, a relationship between doxorubicin exposure and efficacy endpoints could not be fully established, suggesting its limited contribution to the overall effect. Moreover, a negative interaction between lurbinectedin exposure and doxorubicin exposure (AUC*_u_* ·AUC*_DOX_*) was identified. In fact, ATLANTIS suggested that patients who achieved highest exposures for both compounds had shorter survival than the rest. Since the incidence of drug-related adverse events leading to early discontinuation was not higher in this subset of patients, this loss of efficacy could not be attributed to a loss of tolerability.

It is reassuring that the results from the E-R analysis for ORR pointed in the same direction as those for OS, as ORR is an efficacy endpoint that is not affected by confounding factors (e.g., further therapies). A positive relationship between lurbinectedin exposure and ORR was observed in study B-005 as well as in ATLANTIS. However, in ATLANTIS the ORR of patients with low lurbinectedin exposure was higher than the model prediction, although similar to that of patients from the ATLANTIS control arm who received CAV, which may indicate the contribution of doxorubicin to ORR in the experimental arm. On the other hand, ORR of patients with high lurbinectedin exposure in the ATLANTIS experimental arm was lower than that observed in study B-005 at similar lurbinectedin exposures, reflecting the same detrimental effect of doxorubicin observed on OS at high lurbinectedin exposures.

Hence, these findings on OS and ORR may indicate that doxorubicin at higher exposure range did not enhance the activity of lurbinectedin as initially expected, which may explain, at least partially, why addition of doxorubicin to lurbinectedin could not show superiority over the standard of care in ATLANTIS. In this sense, different preclinical studies with the lurbinectedin/doxorubicin combination yielded variable results. An *in vivo* study in mice subcutaneously implanted with different lines of SCLC cells (NCI-H526 and NCI-H82) (18) showed synergistic activity for NCI-H526, while only an additive antitumor activity was found for NCI-H82. Moreover, a set of *in vitro* and *in vivo* experiments used ovarian cancer cell lines treated with lurbinectedin in combination with other antitumor agents (doxorubicin, cisplatin, paclitaxel, and irinotecan metabolite SN-38) (19). The results indicated that lurbinectedin combined with doxorubicin just showed an additive effect while, when combined with SN-38, a strong synergistic effect was observed.

Upon successful development and validation, these models were used to predict alternative dose regimens. Their predictive ability was first confirmed by comparing the predicted vs the observed OS and ORR of the ATLANTIS experimental arm. Then, the efficacy of 3.2 mg/m² lurbinectedin as a single agent was predicted and compared with the observed efficacy in the control arm. An advantage of this approach over indirect comparisons using historical data is that OS and ORR were predicted in the same patients of the ATLANTIS experimental arm, thus allowing a model-based head-to-head comparison of the single-agent full-dose lurbinectedin regimen with the standard therapy for second-line SCLC. The single-agent 3.2 mg/m^2^ lurbinectedin regimen showed superiority over topotecan or CAV for OS (HR: 0.54 [95% CI: 0.41, 0.73]) and ORR (OR: 0.35 [95% CI: 0.25, 0.50]) in the overall population and in patients with sensitive disease (**Figure 3**), confirming the activity of 3.2 mg/m² lurbinectedin in a phase 3 randomized and controlled setting.

A potential limitation of our analysis is that it pooled data from patients with relapsed SCLC from two studies with slightly different exclusion criteria. Patients with brain metastases were excluded in study B-005 and patients with CTFI<30 days were not allowed in ATLANTIS. However, the effect of these characteristics as potential prognostic factors was explored and accounted for during model development. Moreover, the head-to-head comparison was conducted in patients with no brain metastases, providing similar results for OS (HR: 0.43 [95% CI: 0.31, 0.59]) and ORR (OR: 0.30 [95% CI: 0.21, 0.44]). Nevertheless, there could still exist unidentified differences in the distribution of patient characteristics between study B-005 and ATLANTIS, thus limiting the validity of our results. Another potential limitation of our analyses is the type of model used for ORR (sigmoid-E*_max_*) that did not retain any covariate other than AUC*_u_*, and could derive in an overestimate of this efficacy parameter.

In summary, an E-R analysis was used to dissect the individual contribution of lurbinectedin and doxorubicin to the antitumor effects observed in ATLANTIS. By means of this model-based exposure-driven approach, the efficacy of 3.2 mg/m² lurbinectedin as a single agent was predicted. This regimen showed superiority over topotecan and CAV in the overall population and in patients with sensitive and very sensitive disease.

## Data availability statement

The raw data supporting the conclusions of this article will be made available by the authors, without undue reservation.

## Ethics statement

The studies involving human participants were reviewed and approved by Comite de Etica de la Investigación con Medicamentos Hospital Universitario Ramón y Cajal, Madrid, Spain. The patients/participants provided their written informed consent to participate in this study.

## Author contributions

RL and SF contributed to the conception and design of the study. LP-R conducted the analyses and drew the figures. EA-P helped with expert review. SF and RL wrote the manuscript. AZ contributed with a critical assessment. All authors contributed to the manuscript revision. All authors read and approved the final manuscript.
