# Supplementary material for: A model-based head-to-head comparison of single-agent lurbinectedin in the pivotal ATLANTIS Study
Source: Front Oncol. 2023 Jun 15;13:1152371. doi: 10.3389/fonc.2023.1152371 (PMC10311440; doi:10.3389/fonc.2023.1152371)
Supplement: Supplementary file 1 [file DataSheet_1.docx]

Supplementary Material

# PK sampling times, plasma concentration measurement, and OS and ORR definition.

PK samples in study B-005 were collected on cycle 1 and 2, at pre-infusion, 5 min before end of infusion (EOI), and at 0.25, 1, 3, 24, 72, and 168 h after EOI; in ATLANTIS, PK samples were collected on cycle 1 and 2, 3 or 4, at pre-infusion, 5 min before EOI, and at 1 and 95 h after EOI for lurbinectedin, and pre-dose, 5 min after end of administration (EOA), and at 2 and 96 h after EOA for doxorubicin.

Total plasma concentration of lurbinectedin was measured using an ultra-performance liquid chromatography tandem mass spectroscopy assay (UPLC-MS/MS) as described elsewhere (*in press*). For doxorubicin and its metabolite doxorubicinol, total plasma concentrations were measured using a validated UPLC-MS/MS. The lower limit of quantitation of both analytes were set to 1.0 ng/mL. The inter-run accuracy and precision ranged from –9% to 5% and from 6.4% to 20% for doxorubicin, and from –15% to 5% and from 6.0% to 12.9% for doxorubicinol.

Overall survival (OS) was defined as the time from the date of first infusion (in study B-005) or randomization (ATLANTIS) to the date of death or last contact. Objective response rate (ORR) was defined as the percentage of patients with complete or partial response, using RECIST v.1.1 (Response Evaluation Criteria in Solid Tumors; <http://doi.org/10.1016/j.ejca.2008.10.026>). In study B-005, radiological tumor assessments were done at baseline, and every 2 cycles from the onset of the study treatment until cycle 6 or evidence of disease progression. After cycle 6, tumor assessment was performed every 3 cycles until evidence of disease progression. Objective responses had to be confirmed by at least 4 weeks after. In ATLANTIS, tumor assessment was performed every 2 cycles or evidence of disease progression and both confirmed and unconfirmed responses were considered for ORR.

**Supplementary Tables and Figures**

**Table S1. Summary of lurbinectedin pharmacokinetic parameters in the final model and bootstrap results**

|  | Final model | Non-parametric bootstrap | |
| --- | --- | --- | --- |
| Model parameter | Estimate (RSE%) | Mean (RSE%) | Median (95% CI) |
| Parameters |  |  |  |
| V1 (L) | 10.86 (4.16) | 11.6 (4.26) | 11.6 (10.59, 12.56) |
| CL (L/h) | 10.74 (1.77) | 10.7 (1.83) | 10.68 (10.32, 11.08) |
| V3 (L) | 462.2 (2.28) | 460.28 (2.37) | 459.4 (439.23, 483.94) |
| Q3 (L/h) | 15.96 (1.72) | 15.96 (1.75) | 15.98 (15.42, 16.49) |
| V2 (L) | 34.1 (1.85) | 33.3 (1.96) | 33.29 (31.95, 34.57) |
| Q2 (L/h) | 32.38 (2.39) | 30.08 (2.89) | 30.09 (28.24, 31.82) |
| Covariate parameters |  |  |  |
| V1-AAG | -1.18 (7.49) | -1.09 (-8.34) | -1.08 (-1.29, -0.93) |
| V1-BSA | 1.03 (13.66) | 1 (15.05) | 1 (0.74, 1.33) |
| CL-AAG | -0.68 (5.43) | -0.69 (-5.99) | -0.69 (-0.77, -0.61) |
| CL-Albumin | 0.61 (21.07) | 0.6 (26.14) | 0.6 (0.33, 0.88) |
| CL-Moderate inhibitor | -0.14 (25.74) | -0.14 (-28.73) | -0.14 (-0.22, -0.06) |
| CL-Strong inhibitor | -0.33 (18.23) | -0.33 (-21.01) | -0.33 (-0.45, -0.18) |
| V3-AAG | -0.61 (6.7) | -0.62 (-7.49) | -0.61 (-0.7, -0.52) |
| V3-BSA | 2.02 (7.3) | 2.04 (7.35) | 2.03 (1.74, 2.33) |
| V3-Female | -0.29 (9.38) | -0.29 (-8.85) | -0.29 (-0.34, -0.24) |
| Q3-AAG | -0.64 (5.11) | -0.63 (-4.48) | -0.63 (-0.69, -0.58) |
| Q3-BSA | 1.13 (10.34) | 1.17 (9.81) | 1.17 (0.94, 1.4) |
| Q3-Female | -0.25 (8.42) | -0.25 (-8.48) | -0.25 (-0.29, -0.2) |
| V2-AAG | -0.63 (6.93) | -0.63 (-6.47) | -0.63 (-0.72, -0.55) |
| V2-BSA | 0.49 (16.39) | 0.46 (15.91) | 0.46 (0.31, 0.61) |
| Residual error |  |  |  |
| RV | 0.3 (2.14) | 0.31 (2.12) | 0.31 (0.29, 0.32) |
| Intersubject variability |  |  |  |
| V1 (L) | 0.43 (10.15) | 0.17 (31.54) | 0.16 (0.09, 0.29) |
| CL (L/h) | 0.48 (2.89) | 0.23 (6.47) | 0.23 (0.21, 0.26) |
| V3 (L) | 0.4 (4.13) | 0.15 (11.63) | 0.15 (0.12, 0.19) |
| Q3 (L/h)-V3 (L) | 0.66 (7.32) | 0.07 (10.36) | 0.07 (0.06, 0.09) |
| Q3 (L/h) | 0.28 (4.58) | 0.08 (9.9) | 0.08 (0.06, 0.09) |
| V2 (L) | 0.29 (7.44) | 0.07 (16.18) | 0.07 (0.05, 0.1) |
| RV | 0.66 (2.73) | 0.44 (6.14) | 0.44 (0.39, 0.5) |

AAG, alpha-1-acid glycoprotein; BSA, body surface area; CI, confidence interval; CL, clearance; RSE, relative standard error; RV, residual variability.

V1, apparent volume of distribution of central peripheral compartment.

V3, apparent volume of distribution of deep peripheral compartment.

Q3, intercompartmental clearance for deep compartment.

V2, apparent volume of distribution of shallow peripheral compartment.

Q2, intercompartmental clearance for shallow compartment.

**Table S2. Summary of doxorubicin and doxorubicinol pharmacokinetic parameters in the final model and bootstrap results**

|  | Final model | Non-parametric bootstrap | |
| --- | --- | --- | --- |
| Model parameter | Estimate (RSE%) | Mean (RSE%) | Median (95% CI) |
| Parameters |  |  |  |
| CL*_DOX_* | 37.49 (1.45) | 37.36 (1.68) | 37.34 (36.19, 38.57) |
| V4 | 15.64 (2.1) | 15.63 (3.6) | 15.58 (14.61, 16.94) |
| Q7 | 44.95 (1.81) | 44.77 (3.32) | 44.76 (41.97, 47.82) |
| V7 | 1600 (4.28) | 1646.94 (5.93) | 1644.28 (1453.71, 1857.06) |
| Q8 | 22.78 (2.59) | 23.82 (3.58) | 23.81 (22.19, 25.7) |
| V8 | 69.15 (3.65) | 74.32 (3.36) | 74.38 (68.64, 79.17) |
| CL*_M_* doxorubicinol | 152.4 (2.05) | 152.35 (2.2) | 152.38 (145.94, 158.86) |
| V5 | 7734 (2.45) | 7732.83 (2.63) | 7735.27 (7305.74, 8120.61) |
| Residual error |  |  |  |
| RV doxorubicin | 0.31 (4.99) | 0.3 (4.34) | 0.3 (0.28, 0.33) |
| RV doxorubicinol | 0.37 (3.47) | 0.37 (3.14) | 0.37 (0.35, 0.4) |
| Intersubject variability |  |  |  |
| CL*_DOX_* | 0.22 (6.57) | 0.05 (11.81) | 0.05 (0.04, 0.06) |
| Q7 | 0.2 (8.89) | 0.04 (33.58) | 0.04 (0.02, 0.07) |
| V7 | 0.38 (12.61) | 0.16 (26.68) | 0.16 (0.08, 0.25) |
| CL*_M_* doxorubicinol | 0.34 (9.25) | 0.11 (12.41) | 0.11 (0.09, 0.14) |
| V5 | 0.44 (5.6) | 0.19 (9.8) | 0.19 (0.16, 0.23) |
| RV doxorubicin | 0.97 (3.92) | 0.93 (7.36) | 0.93 (0.8, 1.07) |
| RV doxorubicinol | 0.62 (4.64) | 0.38 (9.67) | 0.38 (0.31, 0.46) |
| CI, confidence interval; CL*_DOX_*, clearance of doxorubicin; CL*_M_*, clearance of doxorubicinol; RSE, relative standard error; RV, residual variability.  Q7 and Q8, two intercompartmental distribution clearance of doxorubicin.  V4, apparent volume of distribution of central peripheral compartment of doxorubicin.  V5, apparent volume of distribution of central peripheral compartment of doxorubicinol.  V7, apparent volume of distribution of deep peripheral compartment of doxorubicin.  V8, apparent volume of distribution of shallow peripheral compartment of doxorubicin. | | | |

**Figure S1.**


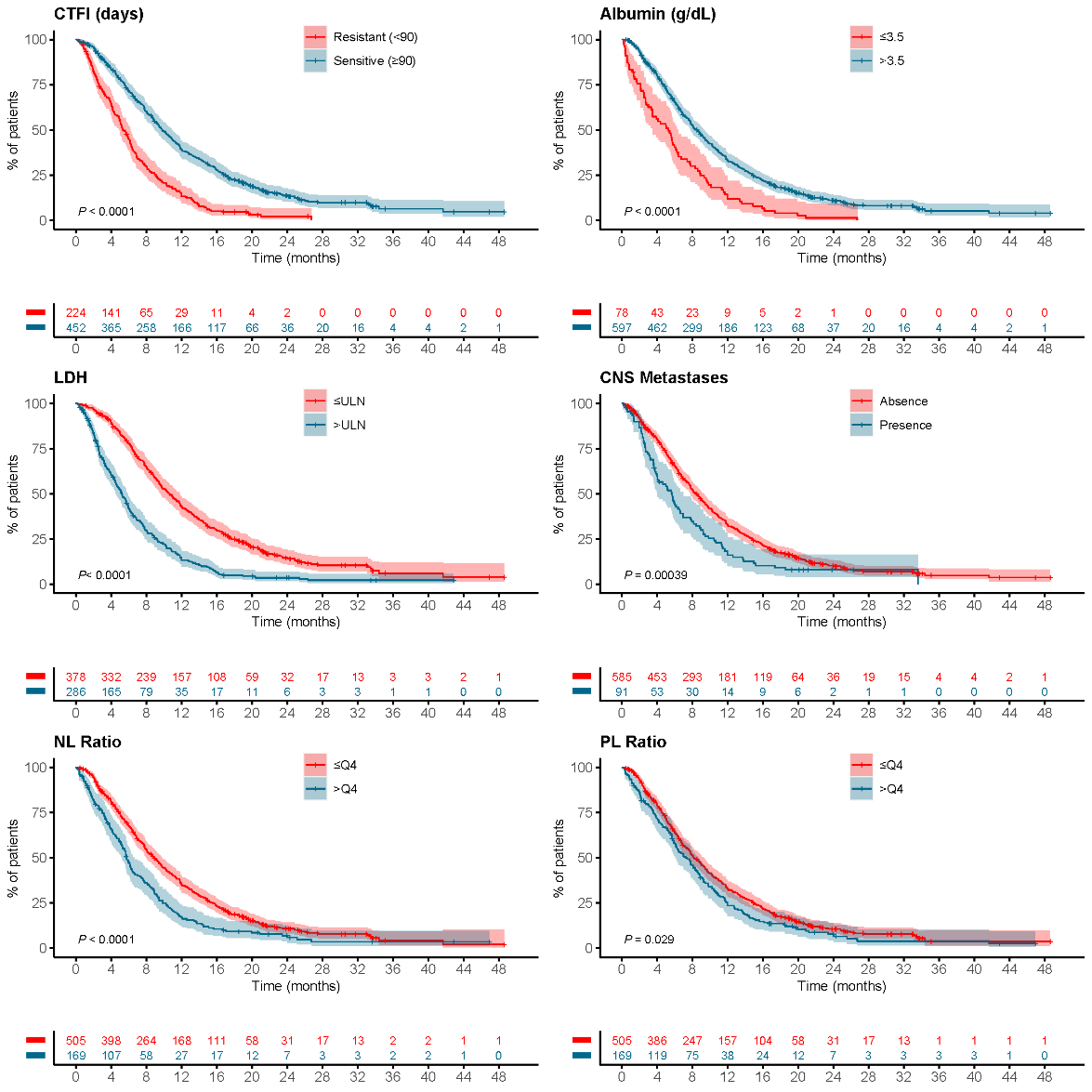


**Figure S1.** Kaplan*-*Meier plots for OS as a function of model predictors in the complete dataset (i.e.*,* study B-005 and experimental and control arm*s* of ATLANTIS).

AAG, alpha-1-acid glycoprotein; CNS, central nervous system; CTFI, chemotherapy-free interval; LDH, lactate dehydrogenase; NL, neutrophil to lymphocyte; OS, overall survival; PL, platelet to lymphocyte; Q4, highest NL ratio/PL ratio quartile; ULN, upper limit of normality.

Note: AAG was not represented in the figure because it was evaluated in the univariate Cox regression analysis only as a continuous covariate, although it showed a p-value <0.001.

**Figure S2.**

**(A)** **(B)**


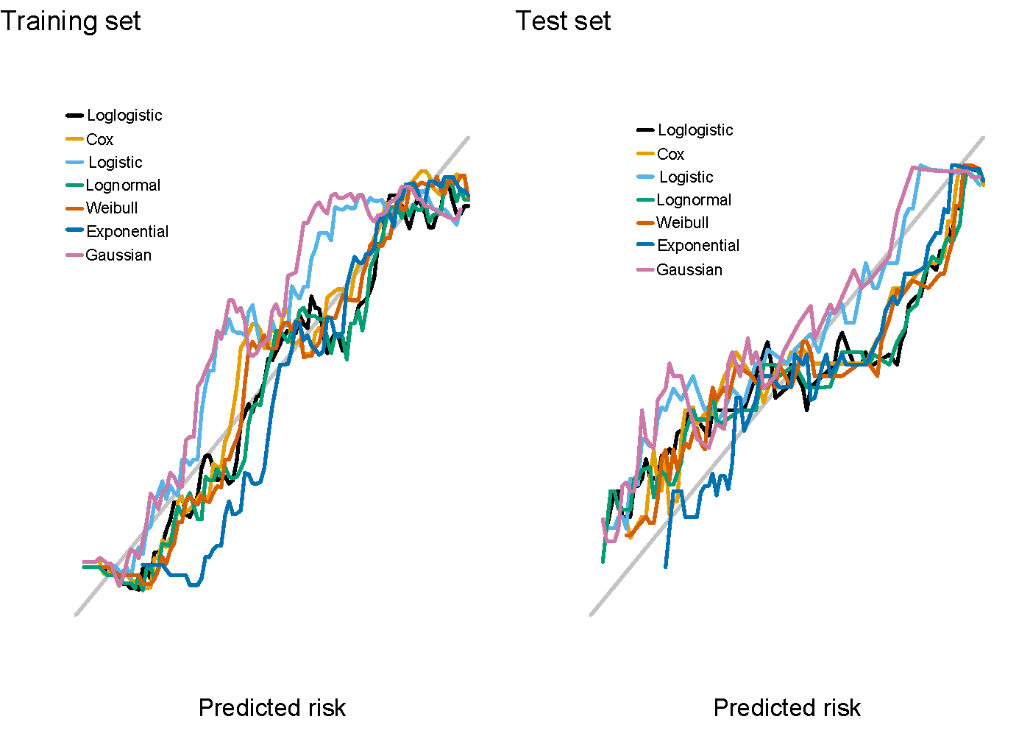


**Figure S2.** Calibration plots of candidate models for OS. Distribution selection in (**A**) the training set and (**B**) the test set. OS, overall survival.

**Figure S3.**

**(A)** **(B)**


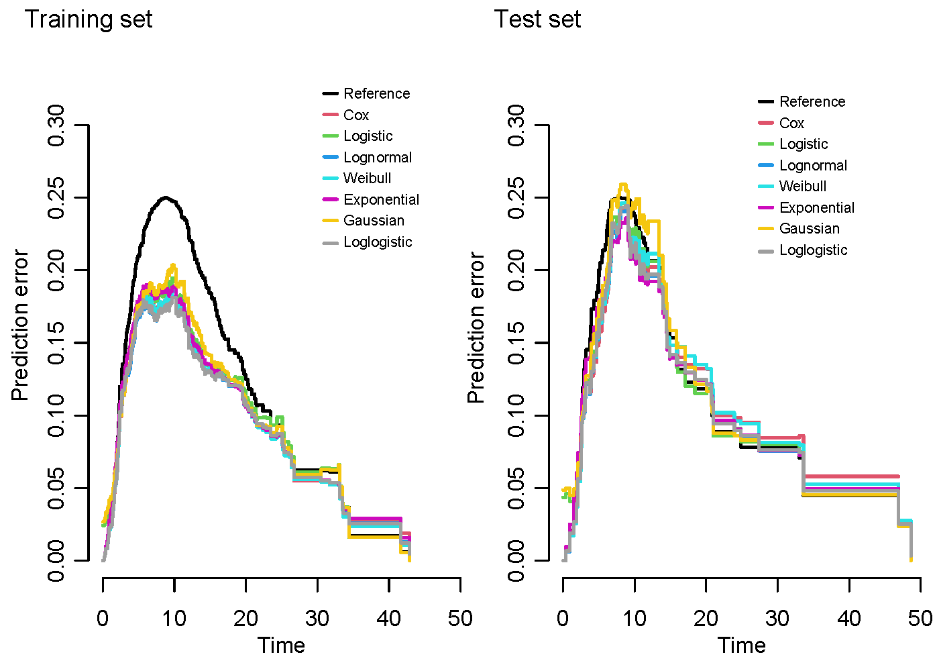


**Figure S3.** Predictive error plots of candidate models for OS. Distribution selection in (**A**) the training set and (**B**) the test set. OS, overall survival.

**Figure S4.**


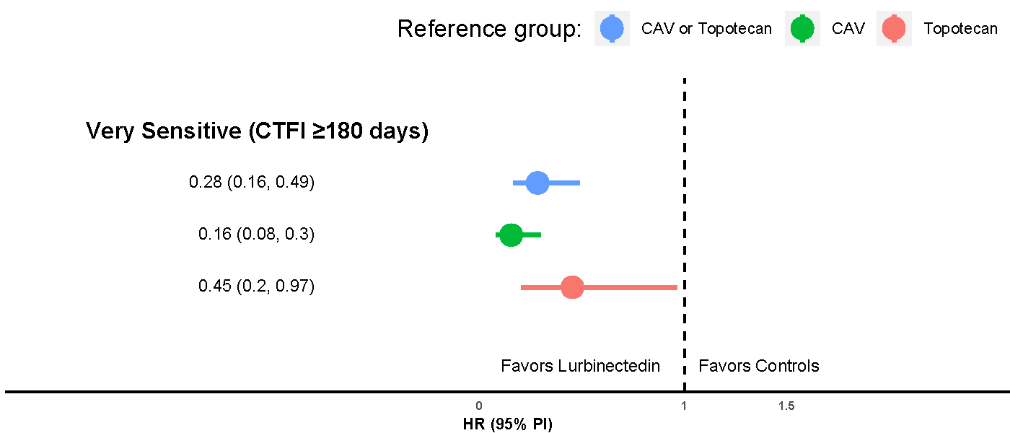


**Figure S4.** Hazard ratios for OS of predicted efficacy with lurbinectedin 3.2 mg/m² as a single agent in ATLANTIS vs the control arm in very sensitive patients (CTFI ≥180 days). CAV, cyclophosphamide plus doxorubicin plus vincristine; CTFI, chemotherapy-free interval; HR, hazard ratio; OS, overall survival; PI, prediction interval.
